# The impact of injury: The experiences of children and families after a child’s traumatic injury

**DOI:** 10.1177/0269215520975127

**Published:** 2020-12-07

**Authors:** Samantha Jones, Sarah Tyson, Janelle Yorke, Naomi Davis

**Affiliations:** 1 Division of Nursing, Midwifery and Social Work, School of Health Sciences, Faculty of Biology, Medicine and Health, University of Manchester, Manchester, UK; 2 Department of Trauma and Orthopaedics, Royal Manchester Children’s Hospital, Manchester Foundation Trust, Manchester, UK; 3 Manchester Academic Health Science Centre, Manchester, UK

**Keywords:** Qualitative study, traumatic injury, paediatric rehabilitation, physical activity, major trauma

## Abstract

**Objective:**

To explore the experiences of children and families after a child’s traumatic injury (Injury Severity Score >8).

**Design:**

Qualitative interview study.

**Setting:**

Two children’s major trauma centres in England.

**Participants:**

32 participants: 13 children with traumatic injuries, their parents/guardians (*n* = 14) and five parents whose injured child did not participate.

**Methods:**

Semi-structured interviews exploring the emotional, social, practical and physical impacts of children’s injuries, analysed by thematic analysis.

**Results:**

Interviews were conducted a median of 8.5 months (IQR 9.3) post-injury. Injuries affected the head, chest, abdomen, spine, limbs or multiple body parts. Injured children struggled with changes to their appearance, physical activity restrictions and late onset physical symptoms, which developed after hospital discharge when activity levels increased. Social participation was affected by activity restrictions, concerns about their appearance and interruptions to friendships. Psychological impacts, particularly post-traumatic stress type symptoms often affected both children and parents. Parents’ responsibilities suddenly increased, which affected family relationships and roles, their ability to work and carry out daily tasks. Rapid hospital discharge was wanted, but participants often felt vulnerable on return home. They valued continued contact with a healthcare professional and practical supports from family and friends, which enabled resumption of their usual lives.

**Conclusions:**

Injured children experience changes to their appearance, friendships, physical activity levels and develop new physical and mental health symptoms after hospital discharge. Such challenges can be addressed by the provision of advice about potential symptoms, alternative activities during recovery, strategies to build resilience and how to access services after hospital discharge.

## Introduction

Injury is a leading cause of death and disability in children worldwide.^1^ The implementation of specialist, centralised trauma services in England in 2012 improved outcomes for severe or life-threatening injuries by focussing on acute care.^2^ Following this success major trauma services are now shifting their attention to enhancing services covering the remainder of the recovery trajectory and longer-term outcomes. Our aim was to explore the experiences of injured children and their families after hospital discharge, to better understand their problems and inform holistic patient-centred service development.

Although there is a body of evidence regarding the effects of a child’s traumatic injury on the child and their family, it has focussed mainly on traumatic brain injuries^3–8^ or adolescents’ and parents’ experiences.^9,10^ Traumatic brain injury is often one of the most serious, but not the most common major trauma injury.^11^ However, there is limited literature concerning the impact of the other types of injuries on children and their families.

This study explored the experiences of injured children and their families after discharge from a major trauma centre. To expand on previous research children with a range of ages and injuries were included.

## Method

This qualitative interview study was conducted between March 2018 and August 2019 at two children’s major trauma centres (specialist centres designated and resourced to deal with the most severe trauma injuries) in England.^12^ It was approved by the National Health Service, North West – Greater Manchester South Research Ethics Committee (REC reference 17/NW/0615) and the Health Research Authority.

### Participants

Admission records were screened against the following criteria:

- Children with all types of injuries (Injury Severity Score > 8) managed in a major trauma centre who were 6–15 years of age at the time of injury, and discharged from the centre within the previous 12 months.
- Parents or guardians aged ⩾18 of children who fulfilled the criteria were also recruited and whose children were aged 2–15 years at the time of injury.

Participants who were discharged less than 2 weeks previously were not approached as they had insufficient experience of being at home. Babies (less than 2 years old) and children with isolated burn injuries, non-accidental injuries or those for whom there were significant safeguarding concerns were excluded.

Potential participants were provided with age appropriate study information by post or in-person by a trauma co-ordinator and invited to take part in the study. Interested parents or guardians spoke to researchers directly about their and/or their child’s participation. The research team provided a further explanation of the study, answered any questions and arranged an interview. Prior to participation informed written consent was taken from parents or guardians and assent from their children. Purposeful sampling was adopted to ensure that a range of injuries, time frames since injury, genders and ages were represented. Recruitment continued until data saturation and maximum variation sampling was reached.

### Data collection

Semi-structured interviews were conducted with the child and parent/guardian together or separately depending on the participants’ preference and what was most appropriate for the child concerned. Participants were given the choice of conducting interviews on the telephone or in-person (in the hospital, participants’ home or neutral location). In-person interviews were recommended for children. However, the option of a telephone interview was offered to parents to minimise participant burden. All interviews were digitally recorded and transcribed verbatim.

One author (SJ, clinical researcher and major trauma rehabilitation coordinator) was fully trained in interviewing children and completed all interviews. A semi-structured topic guide was used to explore the child’s and when appropriate, parent’s or family’s experiences. A copy of the interview guide has been included in the supplementary material; some of the questions included relate to companion papers. Questions in the topic guide were derived from our previous scoping review^13^ and consultation with our service users’ group. Participants were asked about their experiences of health and community services provided after hospital discharge and the social, emotional, physical and practical impacts of the injury. Interviews focused on but were not limited to experiences after hospital discharge. The questions/topics were adapted to suit the age requirements of the child. Parents described both their experiences, those of the injured child and other family members.

### Analysis

Transcribed interviews were anonymised and imported into Excel and NVivo 11 to enable in-depth thematic analysis.^14^ SJ read and re-read each transcript to become familiar with the data and develop potential themes. Themes and sub-themes were based on interview topics and identified through discussion with the research team (SJ, ST and JY). SJ reviewed the full dataset for every participant to identify statements which represented the existing themes and could be coded as such. Some statements led to the development of emergent themes and were added as new sub-themes. An example of a theme which emerged solely from the data is ‘positive impacts’. Regular peer debriefs^15^ were held with SJ, ST and JY to discuss the ongoing analysis, interpretations and alternative explanations for emergent findings. Data within emergent findings was reviewed to check that there was sufficient data to justify new sub-themes. The data was categorised according to the group’s consensus.

## Results

Twenty-six interviews were conducted involving 32 participants; three by telephone and the remainder were in person. Five were conducted jointly with the child and a parent/guardian. Seven child/parent dyads were interviewed separately, and for one child/parent triad the mother and father were interviewed together and their child separately. Five interviews were conducted with mothers alone (i.e. their child did not participate), and three of these were telephone interviews. Interview duration ranged from 11 to 76 minutes (median 29 minutes, IQR 23 minutes). Interviews took place a median of 8.5 months (IQR 9.3) after the injury. Participant characteristics and the interview structure are shown in Table 1. Children described their experiences in varying degrees of detail depending on their age and level of openness, but all accounts contributed to the themed analysis irrespective of detail provided. Data is presented as two overarching themes: (i) the impact of the injury and (ii) experiences of discharge from the hospital and returning home. Each theme has sub-themes detailed in Table 2.

**Table 1.** Participant characteristics.

Interviewee	Gender of child	Child’s age at interview (years)	Injury type	Mechanism	Time post- injury (months)	Interview structure
1. Child	Male	11	Limbs	Road traffic	7	Joint
2. Mother				Incident		
3. Child	Female	12	Multiple*	Fall > 2 metres	10	Joint
4. Mother						
5. Child	Male	14	Multiple*	Road traffic	12	Separate
6. Mother				Incident		
7. Mother	Male	15	Limb	Sport	12	Mother only
8. Mother	Female	5	Limb	Fall < 2 metres	1	Mother only
9. Mother						Separate
10. Child	Female	15	Multiple*	Other	11	
11. Mother	Male	16	Multiple*	Other	11	Mother only
12. Mother	Female	11	Multiple*	Other	11	Mother only
13. Child	Male	11	Abdomen	Road traffic	1.5	Separate
14. Mother				Incident		
15. Child	Female	13	Limbs	Other	12	Joint
16. Mother						
17. Child	Male	13	Head	Fall <2 metres	2	Child separate
18. Mother						(Joint mother & father)
19. Father						
20. Mother	Male	13	Limbs	Sport	11	Mother only
21. Child	Female	13	Limbs	Other	12.5	Joint
22. Mother						
23. Mother						Separate
24. Child	Male	15	Chest and abdomen	Fall < 2 metres	2	
25. Child	Male	10	Abdomen	Sport	2	Separate
26. Mother						
27. Child	Male	8	Abdomen	Fall<2 metres	3.5	Separate
28. Mother						
29. Child	Male	9	Multiple*	Road traffic	2.5	Separate
30. Mother				Incident		
31. Child	Female	13	Spine	Fall < 2 metres	1.2	Joint
32. Guardian						

* Multiple injuries describe two or more injuries affecting different body parts.

**Table 2.** Thematic framework of findings.

Theme	Subtheme
Impact of the injury	Physical and cognitive impacts
	Practical impacts
	Emotional impacts
	Positive impacts
	Impact of family life
	Social impacts
Experience of discharge from hospital and returning home	Adapting to home
	Co-ordinating transition and ongoing care
	Problem-solving

### Theme 1: Impact of the injury

All participants described the child’s injury as a major event which completely disrupted the child’s and family’s life in unexpected ways. As a result of the injury participants reported a wide range of impacts, which are summarised in Table 3. Some physical and cognitive impacts were apparent as an immediate effect of the injury, while many others only came to light after hospital discharge when children returned to school and/or became more physically active:

*‘[he] had probably done slightly more than he can do walking wise. . .So, just really, really tired. And then, that night. . . . maybe the day after, he had some weird hallucinations’.* (Parent 18)

**Table 3.** Summary of injury impacts.

**PHYSICAL AND COGNITIVE IMPACTS OF THE INJURY**
Physical impairments: Mobility/ motor functioning problem
Sensory impairments: Altered, speech, hearing and vision
Physical symptoms: Pain/discomfort, fatigue, breathing difficulties, nausea (vomiting), seizures, headaches, hallucinations, changes in appetite/weight, sleep disturbance, reduced function, mobility, balance and stamina/strength
Physical appearance: Scars, bruises, cuts, hair loss, squints, external fixators on limbs and limps
Cognitive impairments: Altered abilities/behaviour, memory loss, poor concentration
**PRACTICAL IMPACTS**
**Child and parent:** Demand of bureaucracy and attending multiple appointments
**Parent:**Increased care requirements of injured child, adaptation of working patterns, time off work, loss of income, difficultly with daily chores (shopping, cooking, cleaning) and care of the injured child’s siblings. Increased reliance on family members (especially grandparents), and friends for care of the injured child and/or their siblings
**Child:** Increased dependence on parents/family members. Reduced attendance to school
**Emotional impactS**
**Child and parent:** Post-traumatic stress (panic attacks, flashbacks, loss of motivation, low mood, social withdrawal), feeling: upset, guilty, sad
**Child:**Loss of confidence, feelings of self-consciousness, boredom, frustration, anger and increased need for emotional support
**Parent:** Prioritising of child’s needs before their own, fear/worry about injured child’s recovery/future, difficulty watching child suffer, relief when child recovers, ‘emotional rollercoaster’
**POSITIVE IMPACTS**
**Child and parent:**New appreciation of life/friends, reassessment of priorities/life goals, increased empathy for those suffering from illness or injury, wanting to be of service to others
**Child:** Bravery, resilience, determination and increased maturity
**IMPACTS ON FAMILY LIFE**
Changed responsibilities/roles within the family, increased focus on the injured child, reduced contact between family members whilst the injured child is in hospital
**SOCIAL IMPACTS**
**Child:** Changed friendships: strengthened, broken or new friendships with children with injuries/an illness. Friendship difficulties: maintaining contact with friends during recovery/absence from school, difficulty making new friends when an injury occurs during the school transition
**Parent:** Forming bonds with parents in a similar position

Fatigue was one of the most common of these ‘late-onset’ symptoms, but seizures, hallucinations, pain, appetite changes and weight gain associated with inactivity also occurred. These were a frequent cause of concern especially when participants had not been forewarned about them; they didn’t understand why they were happening, nor how to manage them. Participants also explained how physical problems changed over time and were felt to be indicators of recovery.

Injury management in terms of activity restrictions and protective measures had a profound impact on the participants’ lives. Most children described this as boring, annoying and frustrating because they could not participate in the same activities, sports and hobbies as their peers. Furthermore, frustrations relating to activity restriction sometimes progressed and resulted in low mood:

*‘when he couldn’t go out play, this is the part that made.. . ..him feel down’.* (Parent 7)

Participants found more alternative, sedentary activities or devised ways of staying connected to their original hobbies with varying degrees of acceptance. Parents often had to reinforce activity restrictions because children would forget them or didn’t understand their importance. Most parents found the resumption of activities worrying because they didn’t know when it was safe to do so and were anxious about the risks of re-injury:

*‘I’m a bit nervous with him. . .you see he’s not played out yet, I’ve not let him play out yet. I’m a bit, reluctant to do that. . ..obviously it’s just constantly in my mind’.* (Parent 14)

In contrast to their parents, children were largely keen to return to activities, although several children who were involved in more competitive sports reported a loss of confidence.

There were many practical repercussions of the child’s injury which parents had to manage. First and foremost, they had to adjust their working patterns to look after their injured child. Most parents reported that their employers were supportive, with a variety of strategies to manage time away from work including annual leave, sick leave, compassionate leave, unpaid leave, a combination of paid and unpaid leave or working from home. Self-employed parents often found these adjustments relatively easy to make as they had control over their working patterns. However, several parents, particularly the self-employed, suffered a considerable drop in income. Managing on-going healthcare needs after discharge from hospital also had a significant impact on the children and their parents. They faced frequent hospital appointments as they were often treated by several specialists who were located in different departments and ran clinics at different times. Attending appointments often involved lengthy commutes, which affected attendance at school and work, and disrupted daily routines:

*‘It was chaos, because we were still under so many consultants. Even though we were out of hospital. . . .we had to go for all these different appointments’.* (Parent 22)

Dealing with bureaucracy was also highlighted as a challenge, especially the completion of lengthy, complex forms (such as application for disability living allowance) and obtaining sickness certificates.

The injured child and their family experienced a wide range of emotional and psychological impacts which affected them throughout the recovery:

*‘[it’s a] psychological and emotional journey. . .[which] was ..quite emotionally draining and tiring’.* (Parent 18)

Parents reported that they were ‘*exhausted*’ as a result of looking after their injured child and they didn’t have the ‘*energy for anything else*’. (Parent 9) Subsequently, their own needs were compromised:

*‘I quit the gym . . . .because I didn’t want to leave him for too long’.* (Parent 20)

However, children appreciated emotional support, particularly from their parents, who also wanted to fulfill this compassionate role and help their child recover:

*‘me helping him, being with him, supporting him, . . .., I was fine with that. For me it was, like, my son needs me, I need to be there for him and I need to get him better’.* (Parent 7)

The extensive support that participants received from family, friends, professionals and the community in the form of get well wishes, presents, cards, visits or just simply being there helped them know they were cared about:

‘*it feels nice when you have someone or have loads of people who you know are,. . ., thinking about you and hoping you’re okay’.* (Child 17)

The injury impacted on the children’s and parent’s mental health. Many described post-traumatic stress type symptoms, which often only fully developed after hospital discharge. These symptoms often involved flashbacks, which were triggered by specific environments or noises and caused sleep disturbances:

*‘And you had months and months of . . . .being scared of going to bed at night. She was alright through the day, just that fear of dreams, fear of flashbacks. . …’* (Parent 16)

Participants often responded to such symptoms by avoiding places or activities associated with the accident. One child described how she shut herself off from the world. Coupled with this, injured children often felt self-conscious about changes to their physical appearance e.g. scars/external fixators, or having a limp. This led them to avoid social interactions:

*‘Yeah, I eventually had them [friends] round but I’d always like cover it [the injury] up for them and stuff like that, I used to put like [a] blanket over it’.* (Child 21)

Parents also found it difficult to accept changes to their child’s appearance:

*‘he [child’s father] said, I don’t like. . .it’s ruining his looks and all this. He said don’t you want him perfect again?’* (Parent 6)

However, there were some positive impacts. For some participants, the injury brought a new appreciation for life, which changed their approach to it accordingly. They described ‘*living for the today’* and ‘*grabb[ing] every opportunity’*. Their experiences also gave them more insight and empathy to those suffering from illness or injury:

*‘Even though you’re not going to get on with everyone, try and be everyone’s friend . . .. be kind to everyone even if you’re not going to get on. . . .. . ., but at least support them if they’re. . . .going through. . . an injury’.* (Child 17)

One adolescent changed his career plans as a result of the injury, to pursue a caring profession. Many parents also gained new or previously unrecognised appreciation of their child’s bravery, resilience and determination.

‘*. . .the way [injured child] has coped with it has helped us. . . The fact that she can cope with it, then we can cope. If she’s alright, then we’re alright’.* (Parent 9)

All participants recognised that the child’s injury affected the whole family, impacting on their routines, relationships and wellbeing. Spending time together as a family was difficult whilst the injured child was in hospital and thereafter, when they could not take part in the same activities as everyone else. Subsequently, family members felt disconnected from one another:

*‘I was in hospital for ages.. . .I didn’t see like my sister, my dad, my brothers for quite a while. And my pets’.* (Child 1)

The impact on family life was particularly felt when the injured child was discharged from hospital. This was a time when parents experienced a significant increase in responsibilities because their previously independent child now often needed assistance with activities of daily living, home treatments and/or mobility. Several parents compared this to having ‘*a new baby’*. Irrespective of their child’s age parents increased their supervision of the injured child. This was often for practical reasons, such as reinforcing safety advice or monitoring for seizures, but there was also the sense that parents simply wanted to be close to their child and protect them:

*‘I’m just more cautious with him now, like where you’re going, what you doing, you know. I’m constantly right behind him. Yeah, I don’t let him out of my sight’.* (Parent 30)

As a consequence of the injured child’s new needs, siblings often had to shoulder more responsibility and became more independent:

*‘Because he’s the younger one, he’s normally the one that has more help with things, but as the roles were reversed’.* (Parent 23)

Jealousy sometimes developed because siblings perceived that the injured child was the focus of attention. Family, friends and community often rallied around to provide help with everyday chores such as shopping, making meals, child care and transportation to appointments. Grandparents were particularly important because of their close relationships with the injured child and their siblings:

*‘They [child’s grandparents] stepped right into our places and I don’t need to explain anything to them, they know the routines’.* (Parent 28)

The injured children also experienced the impact of the injury from a wider social context. Friends were frequently identified as an important source of support. Therefore, it was important for children to maintain contact with friends, but this was difficult when injuries resulted in a prolonged absence from school or occurred during school transitions:

*‘I think she found it difficult,.. forming bonds and friendships because . . .. she didn’t actually know where she fitted in’.* (Parent 22)

However, friendships often changed, some strengthened whilst others broke down because friends no longer understood the injured child. New, more supportive friendships were often established, occasionally with children they met in hospital, who could relate to their experiences:

*‘She used to check up on me every day. . …cause she.. knows how it feels to. . . .come out of an operation and be like swarmed with messages . . ..and how it felt to be in hospital and not want to do anything. . . she kind of gets it in a way, so we kind of relate to each other’.* (Child 21)

Parents also formed bonds with other parents in a similar position because they ‘*felt a bit of a connection to people who’ve gone through something similar to you’.* (Parent 18)

### Theme 2: Experience of discharge from hospital and returning home

Both the injured children and their parents expressed an overriding need to be discharged from the hospital as soon as possible, but adapted to life at home with varying degrees of ease. Most experienced a sense of relief because they felt more comfortable in their own environment and particularly wanted to *‘sleep in their own bed’*. The return home also represented an opportunity to *‘get back to normal’* and some participants made immediate adjustments, whilst others took longer to adapt:

*‘we kind of became institutionalised. Yeah, so it’s not [laughs] the hospital keeping us hostage, but it’s that Stockholm syndrome thing where, you know. . .. you prefer being in hospital, ‘cause it was what we knew’.* (Parent 18)

Families wanted to maintain the closeness they had in hospital by continuing to sleep in close proximity at home, for example:

*‘The next thing I knew [injured child] had crept in beside me . . . it’s suddenly insecure to be home’.* (Parent 19)

Some parents felt vulnerable away from the hospital environment and described the discharge home as daunting and frightening. They described the hospital as ‘*security blanket’* or a ‘*bubble’* where they knew their child was safe. Several parents only fully appreciated their child’s needs and what would be involved in looking after them once they were home.

Most talked about aspects of care that helped or hindered the transition from hospital to home. Support was needed to co-ordinate the transition and to provide on-going aftercare so that families could ‘*just concentrate on being with [their injured child] and adjust to being at home’* (Parent 4). Parents felt that managing these aspects of care were too difficult to organise themselves as they did not know what was needed. Therefore, having continued access to healthcare professionals was important. Participants felt reassured when they knew there was someone to answer their questions which frequently only arose after discharge:

*‘Knowing that we could ring up about anything, no matter how silly it might seem’.* (Parent 11)

When co-ordination failed or was not provided, participants felt abandoned by healthcare services and had to solve problems independently:

*‘the whole focus in hospital is to get people out. And then once they’re out. . ..it kind of stops.* (Parent 22) *Because everyone just sort of kind of assumes you’re better, don’t they?* (Child 21) *There’s nothing. You’re on your own’.* (Parent 22)

The type of problems participants experienced included equipment not being delivered, follow-up appointments not being organised, difficulty accessing services or not knowing which services had been approved. In such circumstances, parents had to advocate for their child. They explained that:

*‘[better co-ordination] would just have eased the journey for us. . . .by not having to constantly battle to get stuff that you should have got in the first place’.* (Parent 8) Consequently they had to take action: *‘She wasn’t referred. So I basically did my own referral to the community nurse’.* (Parent 8)

As well as advocating and being proactive in helping their child to access services, parents often found their own innovative solutions to problems. For example, one mother explained how she enabled her child with restricted mobility to have more freedom: ‘*we bought him a mobility scooter. . … . .and it was just easier to get him out the house in the sunshine’.* (Parent 20)

## Discussion

This study found that injured children were particularly affected by changes to their appearance, physical activities, friendships, psychological wellbeing and ‘late onset’ symptoms which only became apparent after discharge (e.g. fatigue). As a result of their child’s injury, parents’ caring responsibilities and family burden increased.^4,9,10,16^ Juggling this with work commitments was a key practical challenge.^9^ They found that flexibility on their and their employers’ part was essential to address these new demands. Adult trauma services often use return to work as an indicator of recovery.^17^ Similarly, in children’s trauma management parents’ return to work may be a useful indicator of recovery and the family’s return to ‘normal’.

One of the key reasons that parents needed to work flexibly was frequent out-patient appointments which were immensely time- consuming.^16,18,19^ This also limited children’s return to school and overall attendance. More accessible and convenient ways of providing on-going care are needed. Possible solutions may lie in the development of virtual clinics, ‘one-stop shop’ multi-disciplinary and/or multi-specialist clinics, or professional input to co-ordinate appointments.

Regardless of the type of injury, the challenges faced by injured children and their families were similar and centred on psychosocial factors. Thus, children with traumatic injuries may be most effectively managed with a biopsychosocial model of care.^20,21^

Unlike studies of children’s TBI (which is often considered a hidden injury),^4^ many of our participants had visible signs of injury which had a profound impact particularly on self-confidence. Most previous research relating to sudden changes to physical appearance involves children with burn injuries, who describe similar experiences.^22–25^ Although, in comparison the degree of physical change may be considered less pronounced or more transient in some of ‘our’ injured children. Nevertheless changes to physical appearance mark an injured child as being different to their peers and may provoke questions or reactions that are difficult to manage.^25^ Healthcare professionals need to be more aware of the impact these changes may have, even those that may be considered relatively minor or temporary. Support is needed to help children develop coping strategies to deal with other people’s reactions and facilitate social integration,^26^ including the maintenance and development of new friendships.

In contrast to previous research on children’s TBI, which focusses on cognitive, behavioural and social impacts, rather than physical problems,^13,27–29^ we found that the physical impact of injury was a major issue, most notably physical activity restriction and resumption. The injured children often felt bored and wanted to play with their peers and return to their usual activities, but parents prioritised protecting their child from further harm and were much more cautious about the return to activity. This apparent tension between parents and children could be eased by providing clear, consistent information and ongoing support from healthcare professionals to manage activity restriction or resumption. Advice about alternative types of play or ways to remain involved with their peers may help to optimise mental and physical wellbeing, which contribute to the development of resilience.^30^ This is an area that would benefit from therapy input, but approaches may vary because the evidence about the return to activity after some types of childhood injury is limited. Clinical guidelines were published almost two decades ago,^31^ with negligible further evidence produced since then. Consequently, clinical advice is often based on tradition, personal preference and clinical experience. Further research is required to develop injury-specific evidenced-based guidelines for children’s return to activity and to test the feasibility and acceptability of clinical recommendations based on clinical opinion.

Again, regardless of the type of injury, both injured children and parents frequently experienced symptoms of post-traumatic stress, which became most apparent after hospital discharge.^32^ This indicates the need for early information on management strategies. In addition to longer-term physical and mental health screening for both child and family with signposting to appropriate psychological services whenever difficulties occur.^19^ It is important to ensure parents’ mental health is addressed promptly to enable them to support their child. Injured children rely heavily on their parents for emotional and practical support during recovery.^9^

Although our participants outlined many challenges, they also demonstrated resilience by adopting strategies to support their wellbeing, such as utilising positive emotional support from family and friends, a problem-solving approach and a positive outlook. Consistent with research on other significant traumatic events, the participants’ community played an important role in helping them to make positive adaptations to return to daily life and work.^30,33^ Future research needs to address how communities can help injured children and their families to develop self-efficacy and resilience.

Specialist trauma services in the UK are required to complete pre-discharge assessments of patients’ rehabilitation needs (referred to as a rehabilitation prescription).^34^ This study indicates that such assessments should evaluate biopsychosocial issues as well as access to practical and emotional support from family, friends and the community. They also need to identify those without such informal networks who may require additional support. As many problems only become apparent after discharge, needs should be monitored throughout recovery. Whilst both these interventions require additional resources, early identification of problems may help to offset the potential costs of the long-term use of health and social care services.^35,36^

We acknowledge some strengths and limitations of this study. Most of the parent participants were mothers. It is possible that different perspectives may have emerged if more fathers or extended family members had been included. Joint interviews or the presence of parents may have influenced the data obtained, both positively and negatively. However, the depth of data is likely to have been enhanced by using participants’ preferred interview format and parent’s insightful prompts during children’s interviews.^37^ A key strength is that the sample has included children with a wide range of ages and types of injury. We believe it is broadly representative of injured children managed by major trauma services,^11^ although excluding babies and those with safeguarding issues. To our knowledge this is the first study to combine both children and parents’ views from a broad cohort of injured patients. Previous studies have predominately included parents ^4,5,9,10^ specific age groups ^19,38–41^ or specific injuries.^3–8^

**Clinical messages**

- Injured children and families are affected by altered abilities, activities, appearance, friendships, roles, responsibilities, and practical demands.
- Post-discharge injured children may develop new symptoms (especially fatigue) which require ongoing monitoring, advice and support.
- Throughout recovery, injured children and families need advice about how to alter, and how/when to resume activities.

## Supplemental Material

sj-pdf-1-cre-10.1177_0269215520975127 – Supplemental material for The impact of injury: The experiences of children and families after a child’s traumatic injury Click here for additional data file. Supplemental material, sj-pdf-1-cre-10.1177_0269215520975127 for The impact of injury: The experiences of children and families after a child’s traumatic injury by Samantha Jones, Sarah Tyson, Janelle Yorke and Naomi Davis in Clinical Rehabilitation
