# Supplementary material for: The impact of injury: The experiences of children and families after a child’s traumatic injury
Source: Clin Rehabil. 2020 Dec 7;35(4):614–25. doi: 10.1177/0269215520975127 (PMC8027929; doi:10.1177/0269215520975127)
Supplement: sj-pdf-1-cre-10.1177_0269215520975127 – Supplemental material for The impact of injury: The experiences of children and families after a child’s traumatic injury [file sj-pdf-1-cre-10.1177_0269215520975127.pdf]

SUPPLEMENTARY MATERIAL

	**Guide to Questions**
**Generic needs**	Tell me about what was different for **you/ you and your child** following your injury?<br><br>What help did ***you/your child*** need after your injury?
**Discharge specific needs**	What was it like for ***you/ you and your child*** when you went home from the hospital?<br><br>When ***you/your child*** went home from the hospital how did you feel?<br><br>Did you feel ***you/you and your child*** prepared to go home?<br><br>Was there anything that worried ***you/ you and your child*** about going home?<br><br>Did ***you/you and your child*** need any support/help from hospital staff/health professionals when you went home from the hospital?<br><br>Did you feel that you got the help ***you/you and your child*** needed?<br><br>Did ***you/your child*** have any difficulties when you went home from the hospital?<br><br>Was there anything that really helped or made things easier when **you/your child** went home?<br><br>**Probes:** Equipment, modifications to home, home visits, advice leaflets, meetings before discharge, follow up appointments, phone calls from the hospital.
**Key Worker**	Did ***you/your child*** have a key worker (a health professional/nurse/physio/occupational-therapist/carer/doctor) who provided help or advice when you went home from the hospital?<br><br>**If answers yes:** Did you find this helpful? Why was this helpful?<br><br>What did the key worker do for you?<br><br>**If answers no:** Do you think that a key worker would have been helpful?<br><br>What help/support could have they provided?
**Information needs**	Was **your/your child's** injury explained to you in the hospital?<br><br>Did you understand the explanation of ***your/your child's injury***?<br><br>Did you understand how the injury would affect ***you/your child?***

	Were you provided with advice about **what you/your child** was allowed and not allowed to do following ***your/your child's injury?*** Was this easy to understand?<br><br>Did you receive any advice or information when you were discharged from the hospital? Probe: information booklet, helplines, follow up appointments/ follow up phone calls.<br><br>**If answers yes:** Was the information helpful/how? Were you given enough information? Was it easy to understand? Who gave you the information?<br><br>**If answers no:** Was there you wanted to know when you went home from the hospital, which you were not told? Was there anything you had to find out for yourself?
**Educational needs**	How did ***you/your child*** deal with returning to school after their injury?<br><br>Did **you/your child** have any difficulties returning to school?<br><br>Did **your/your child's teachers** know that you/your child had suffered from an injury?<br>**Probes:** Did they understand the injury/do anything differently?<br><br>Was there anything that really helped **you/your child** when you returned to school?<br><br>Were there any changes made or advice which made **your/your child's** return to school easier?
**Social needs**	Did **your** friends/family know about **your/your child's** injury?<br><br>Do you think they understood what had happened to ***you/your child***?<br><br>Were ***your friendships*** different in any way following ***your/your child's*** injury?<br><br>Did your friends and family help ***you/your child after your child's injury***?<br><br>Did ***your/your child's*** hobbies/play/sporting activities change following your injury?
**Physical**	Did **you/your child** have any physical problems following the injury?<br><br>(Probes: will depend on the age of child: difficulties walking, talking, crawling, eating, speaking hearing, toileting, returning to their usual activities)<br><br>Did **you/your child** need crutches/walking frame/wheel chair after your injury?

	Did ***you/your child*** have any treatment from therapists /health professionals to help with the physical problems after the injury?<br><br>Therapists and Health Professionals are: physiotherapists/occupational therapists/dieticians/speech therapists/nurses/doctors.<br><br>Did you need additional care/help at home? Probe: adaptions to the home, downstairs living.<br><br>Was there any change to **your/your child's** appearance after their injury?<br><br>**Probes:** scars, cuts & bruises, weight gain or loss, items your child had to wear: supports, casts, brace, breathing pipe.
**Psychological**	Were ***you or your child*** scared or worried after ***your/your child's injury?***<br><br>Did Y***ou or your child*** have any problems sleeping following their injury?<br><br>Did ***your child's/your behaviour*** change following the injury?
**Emotional needs**	How did you feel after ***your/your child's injury?***<br><br>Did ***your/your child's*** injury affect you emotionally?<br><br>Did you receive any emotional support from staff at the hospital or people in your local community?<br><br>**Probes:** worried, concerns for the future, upset, scared.
**Family/work needs?**	Did **your/your child's** injury affect the family or family life?<br><br>***Probes:***<br><br>• Was there any change to the daily routine?<br>• Was there any change to roles/responsibilities within the family?<br>• Do you have other children? Was it difficult to look after them at the time of your child's injury?<br>• Were you working at the time of your child's injury? Did their injury affect work in any way?<br>• Was your place of employment supportive after your child's injury?<br>• Did you need any support to look after your child ?
**Current situation**	What are things like for **you/your child now?**<br><br>Are you receiving help from either the hospital or your community services?

	Have you/ your child regained their previous level of activities at home?<br><br>And at school?
**Unmet needs/met needs**	Looking back over the time since the injury, is there anything that could have been done differently to help ***your/your child's*** recovery?<br><br>With hindsight were there any services which you did not receive which you think would have helped you and your child/you?<br><br>What really helped you after your injury/ you and your child after your child's injury. Probes: Advice/information/people (health professionals/family, friends/people in the community), equipment, support groups, follow ups.
**Closing**	Thank you for much for talking to me today. Do you have any questions or is there anything else you would like to tell me which we haven't covered?
